# Sex Differences in Biological Processes and Nitrergic Signaling in Mouse Brain

**DOI:** 10.3390/biomedicines8050124

**Published:** 2020-05-15

**Authors:** Igor Khaliulin, Maryam Kartawy, Haitham Amal

**Affiliations:** Institute for Drug Research, School of Pharmacy, Faculty of Medicine, The Hebrew University of Jerusalem, Jerusalem 9112102, Israel; igorkh@savion.huji.ac.il (I.K.); maryam.kartawy@mail.huji.ac.il (M.K.)

**Keywords:** sex, proteomics, S-nitrosylation, nitric oxide, posttranslational modification, cell signaling, brain disorders, systems biology, computational biology

## Abstract

Nitric oxide (NO) represents an important signaling molecule which modulates the functions of different organs, including the brain. S-nitrosylation (SNO), a post-translational modification that involves the binding of the NO group to a cysteine residue, is a key mechanism of nitrergic signaling. Most of the experimental studies are carried out on male animals. However, significant differences exist between males and females in the signaling mechanisms. To investigate the sex differences in the SNO-based regulation of biological functions and signaling pathways in the cortices of 6–8-weeks-old mice, we used the mass spectrometry technique, to identify S-nitrosylated proteins, followed by large-scale computational biology. This work revealed significant sex differences in the NO and SNO-related biological functions in the cortices of mice for the first-time. The study showed significant SNO-induced enrichment of the synaptic processes in female mice, but enhanced SNO-related cytoskeletal processes in the male mice. Proteins, which were S-nitrosylated in the cortices of mice of both groups, were more abundant in the female brain. Finally, we investigated the shared molecular processes that were found in both sexes. This study presents a mechanistic insight into the role of S-nitrosylation in both sexes and provides strong evidence of sex difference in many biological processes and signalling pathways, which will open future research directions on sex differences in neurological disorders.

## 1. Introduction

Among the numerous signaling molecules of the central nervous system (CNS), nitric oxide (NO) occupies a special place [1]. NO is produced in the cells from l-arginine by three NO synthase (NOS) isoforms: neuronal NOS (nNOS), inducible NOS (iNOS) and endothelial NOS (eNOS) [2]. nNOS is constitutively expressed in the cytosol of neurons. eNOS is bound to the membranes of endothelial cells. Activity of both nNOS and eNOS are Ca^2+^-dependent. Meanwhile, iNOS is located in the cytosol of glial cells and its activity is independent of Ca^2+^ [3,4,5]. nNOS forms a complex with *N*-methyl-d-aspartate receptor (NMDAR), post synaptic density protein-95 (PSD-95) and PSD-93 [6]. Once the NMDAR is activated by extracellular stimuli, it triggers Ca^2+^ influx. The intracellular Ca^2+^ forms a complex with calmodulin, which prompts the NO formation by nNOS and activation of guanosine monophosphate (GMP) cyclase [7]. In the physiological conditions, NO plays a role of a signalling molecule, taking part in the regulation of various functions. In the CNS, it regulates synaptic plasticity, synaptic activity, and vesicle trafficking.

A key mechanism of nitrergic signaling is S-nitrosylation (SNO). SNO represents the incorporation of a nitrosogroup into a reactive cysteine thiol leading to the formation of a nitrosothiol group [8,9]. At some cysteines, it can be driven by a putative “nitrosylation motif” in the primary structure of nitrosoproteins [8,10]. Such motifs could be incorporated into the three-dimensional structure of cysteines rather than in the primary structure [11]. SNO plays a signaling role in regulation of protein localization, axonal transport, synaptic plasticity and various neuronal pathways [12,13].

However, the overproduction of NO associated with increased NOS activity leads to toxic effects and cell death due to nitrosative stress [13]. In these conditions, NO interacts with superoxide radical and forms peroxynitrite, a highly reactive molecule, which breaks down DNA, lipid and protein [14]. Nitrosative stress is also manifested in the enhanced protein S-nitrosylation (SNO), tyrosine nitration, and S-nitrosoglutathione (GSNO) formation [15,16,17,18]. The aberrant protein SNO participates in progression of many neurodevelopmental, neurobehavioral and neurodegenerative diseases and the processes of senescence [13]. Therefore, studies of the NO and SNO signaling may lead to the discovery of the new molecular targets for treatment of the different neurological disorders.

It should be noted that most of experimental studies are carried out on male animals. However, the evidence of significant sex differences in the NO production and oxidative/nitrosative stress in different brain regions has been accumulated and these differences were determined by both sex hormones and genes. Furthermore, some scientists consider NO as an effector molecule in the determination of sex differences in development [19]. A study on young adult homozygous nNOS-deficient mice revealed that estradiol exerts a positive control on hippocampal NO production *via* estrogen receptor-β–mediated neuronal NOS expression, whilst low estrogen in the female hippocampus corresponds to lower local NO than in the male hippocampus [20]. In that work, stress promoted glucocorticoid-dependent NO production in the hippocampus of males. However, in females, stress suppressed NO production because of decreased estrogen [20]. Sex differences in nNOS mRNA were found in the adult preoptic area/anterior hypothalamic region of rats [21] and mice [22]. In the experiments on rats, Rodriguez et al. did not find any sex difference in the expression of NOS, level of NO and its metabolites in the outer medulla in normal conditions. However, renal ischemia/reperfusion in that study significantly increased NO levels and dimeric/monomeric eNOS and nNOS ratios in females compared to males. At the same time, increases in peroxynitrite current and 3-nitrotyrosine concentration were lower in females than in males. The authors explain these results by the reduced inactivation of NO, released from cellular stores, by peroxynitrite [23]. Studies on the cultured XX and XY rat neurons and 17-day-old rats also showed much higher resistance to oxidative/nitrosative stress in females than in males [24]. Interestingly, it has been shown that this phenotypic difference in the brain appears to be independent of gonadal phenotype [24,25]. Nevertheless, this does not exclude the effects of the gonadal hormones on the sex differences in resistance to oxidative/nitrosative stress. Thus, Raficov et al. have recently reported that the male gender in mice is associated with higher production of oxidants and lower activity of the antioxidant system [26] due to the effects of testosterone and these results were in accord with others showing the role of this hormone in oxidative stress [27,28].

A few proteomics studies on the sex differences in the brain has been carried out previously. Thus, in the samples from the contralateral and the ipsilateral brain areas of male mice subjected to the middle cerebral artery occlusion (MCAO), the altered expression of eight proteins was identified. Among them, the only up-regulated protein in the ischemic area was dihydrolipoyllysine-residue acetyltransferase. In contrast, in the MCAO-affected female samples, SH3 domain-binding glutamic acid-rich-like protein, hypoxanthine-guanine phosphoribosyltransferase, transcriptional activator protein pur-alpha and dihydropteridine reductase were downregulated [29]. Estrogen signaling results in activation of mitogen-activated protein kinase (MAPK) followed by the activation of the cAMP/protein kinase A/cAMP response element-binding protein and phosphoinositide 3-kinase/protein kinase B (Akt) protective pathways [30]. The proteomics and transcriptomics study of the sex differences in microglia has demonstrated a higher protein expression of purinergic receptors and an increased potential to respond to the stimuli like ATP in male mice compared to females. Myosin-related proteins contributing to cell stability, trafficking, shape, and size, as well as the proteins related to Toll-like receptor pathways were also increased in male microglia. One of the proteins enriched in female microglia was Irf3, which could be related to a higher potential to activate type I interferon-related processes. At the same time, the authors found higher expression of Inpp5d and mTOR indicating changes toward a shorter lifespan of male microglia [31]. In total, 1109 genes appeared to be differentially expressed in males and females in the hippocampus and 55 were differentially expressed in the cortex. They identified 72 genes overexpressed and 27 expressed at a lower level in the cortex of male *vs*. female microglia. Among the overrepresented genes in male microglia, Gene Ontology (GO) analysis revealed the increased “transcription factor activity”, “insulin receptor pathway” and “ATP binding”. GO gives an ontology of definite terms that represent gene product properties. The ontology includes three different domains: Cellular Component, Molecular Function, and Biological Process. GO enrichment analysis is to perform enrichment analysis on genes sets of interest. The GO terms “gamma-aminobutyric acid (GABA) and Glutamate receptor activity”, “ubiquitin protein activity” and “magnesium ion transport” were overrepresented in the female microglia [31]. Another transcriptomics study of the developing mouse hippocampus identified a total of 198 transcripts differentially expressed between females and males [32]. The scientists also detected 69 transcripts with complex and sex-specific patterns of temporal regulation through postnatal development. Eight of them were heat-shock proteins. GO analysis of the sexually dimorphic transcript set showed enrichment for the terms “protein folding” and “response to misfolded protein”. These transcripts possessed stable expression in females, but were progressively up-regulated in males, reaching a higher level compared to females at 4 months of age [32].

Considering the importance of NO and SNO in regulation of the brain development and functions in norm and disease, it is critical to study the sex differences in S-nitrosylation of different proteins in the brain. Here we investigate the differences in SNO and on the NO-related biological functions in the cortices of male and female mice in normal conditions. The present work revealed for the first time SNO-related significantly higher enrichment of the synaptic processes in the brain of female mice compared to males, whilst the brain of male mice was characterized by a significant enrichment of cytoskeletal processes. This study is a part of an ongoing project on the role of NO and SNO in the brain functions and signaling pathways in physiological and pathological conditions. The investigation of these processes in other brain regions, including the hippocampus and striatum is currently underway.

## 2. Material and Methods

### 2.1. Materials and Reagents

For Mass Spectrometry (MS), protease inhibitor cocktail, acetonitrile (ACN) and distilled water were purchased from Sigma-Aldrich (St. Louis, MO, USA). Sequencing-grade modified trypsin was provided by Promega (Madison, WI, USA). Vivapsin 10 kDa molecular weight cut off (MWCO) filters were procured from Sartorius Stedim Biotech GmbH (Göttingen, Germany). Biotin-PEG3-propionic acid was obtained from Chem Pep Inc (Wellington, FL, USA). SNOTRAP-biotin synthesis and Nuclear magnetic resonance (NMR) analysis were performed as described previously [33]. For High–Performance Liquid Chromatography (HPLC) and Liquid Chromatography–Mass Spectrometry (LC-MS), HPLC grade solvents were used.

### 2.2. Sample Preparation for MS

Cortex brain tissues were isolated from 6–8-week-old mice. The corpus callosum, limbic system, and cerebellium were not included in these brain samples. The cortex samples were immediately transferred into liquid nitrogen for storage at −80 °C for further analysis. For each biological replicate, 3–4 cortex tissue samples from 3–4 mice were pooled. Further, tissues were homogenized on ice in freshly prepared lysis buffer: 250 mM HEPES-NaOH, 0.1 mM neocuproine, 1 mM EDTA, 1% NP-40, 20 mM iodoacetamide (IAM), 1% protease inhibitors cocktail, pH 7.7. The homogenates were centrifuged (12,000–13,000× *g* for 10 min at 4 °C), the supernatant was collected and protein concentration was estimated by Bradford assay (Bio-Rad, Hercules, CA USA, Cat. No. 500-0006). Next, in the presence of 2.5% SDS, samples were alkylated with 30 mM IAM in the dark at 37 °C. After alkylation, samples were washed twice with 3 times volume of 8 M Urea (in 50 mM HEPES, pH 7.7) and once with 50 mM HEPES (pH 7.7) by centrifugation at 5000× *g* for 30 min at 4 °C with 10 K MWCO spin filters pre-rinsed once with water (Satorious Stedim Biotech GmbH, Göttingen, Germany, Cat. No. VS15T01). After the centrifugation, SNOTRAP labeling stock solutions prepared in 50% ACN were added to all samples to make a final concentration of 1.25 mM in order to convert SNO to stable disulfide-iminophosphorane. Then, all samples were incubated at 25 °C in SNOTRAP solution for 1.5 h. Following the SNOTRAP labeling, reagents were washed out by three consecutive washing using 50 mM HEPES buffer (pH 7.7) with 10 K filters. Afterwards, each sample was incubated with gentle shaking with 200 μL Streptavidin agarose beads (Pierce, Cat. No. 20349) for 1 h at 25 °C. The beads were washed with washing buffer containing: 50 mM HEPES, 150 mM NaCl, 0.1% SDS, pH 7.7 three times followed by three times wash with the buffer containing: 50 mM HEPES, pH 7.7. After washing, proteins were eluted using the buffer containing: 10 mM TCEP in 50 mM HEPES, pH 7.7 and alkylated with 10 mM IAM. Then, protein samples were trypsinized (Promega, WI, USA, Cat. No. V5111) at 37 °C for 4 h and desalted using C18 StageTips as described previously [34]. All samples were prepared at 25 °C in the dark.

### 2.3. MS Analysis

Two biological replicates were run. Each biological replicate consisted of 4 cortex tissue samples. Thus, the cortex samples were extracted from 8 mice in total. Three technical replicates from each biological replicate were pooled. The mass accuracy was preserved using ion *m/z* 1221.9906 as an internal reference. Agilent MassHunter Workstation software was used for the data acquisition.

The digested peptides were analyzed using 6550 Nano-HPLC-Chip/MS system of Agilent, coupled with a micro-autosampler, pumps of a capillary and nanoflow, and the Chip-Cube connected to the LC modules and the MS instrument. H_2_O with 0.1% formic acid (FA) was used as a mobile phase A and ACN with 0.1% FA was used as a mobile phase B. Polaris-HR-Chip-3C18 HPLC-Chip (Agilent Technologies, Cat. No. G4240-62030) separated the peptides. It consisted of a 360 nL enrichment column, a 75 μm × 150 mm analytical column and a 3 μm stationary phase. The peptides were loaded onto the enrichment column. The gradient was set for 55 min, starting from 3% B at 300 nL/min, increased to 30% B and kept from the 2nd to 35th min, then increased to 60% B at the 40th min, to 90% B at the 45th min and then kept stable for 5 min followed by a 5 min after-run at 3% B. We acquired the positive-ion MS spectra using 1700 Da extended dynamic range mode: ESI capillary voltage was set on 1960 V; fragmentor on 360 V; Octopole RF peak on 750 V; drying gas on 13 L/min; drying temperature on 225 °C. The data were acquired at the rate of 6 MS spectra/second and 3 MS/MS spectra/second in the range of *m*/*z* 300 to 1700 for MS and 50 to 1700 for MS/MS. The Max number of precursors per cycle was set at 20, setting the threshold at 5000 ions in a precursor abundance-based scan speed in peptide isotope model with plus 2, plus 3 and above charge-state preference, and with active exclusion after 1 spectrum and released after 0.15 min. The fragmentation energy was set at a slope of 3.1 V/100 Da, including a 1.0 offset for doubly charged precursors, 3.6 V/100 Da with a −4.8 offset for triply and also multiply-charged precursors.

### 2.4. MS Data Extraction and Processing

We used the following parameters for data extractions: precursor MH+ 300–8000 Da, scan time range from 0 to 200 min, cysteine carbamidomethylation for fixed modification, sequence tag length of >1, default for precursor charge, true for find 12C precursor, merge scans with the same precursor at +/−30 s and +/−0.05 *m*/*z*, and MS noise threshold of 100 counts. MS/MS spectra were searched against the mouse SwissProt protein database with +/− 20 ppm precursor ion tolerance and +/−50 ppm fragment ion tolerance. Different modifications of methionine oxidation, deamidation of asparagine, and fixed modification of cysteine carbamidomethylation were included. The generated false discovery rate (FDR) was set to 1.2% for both peptide and protein identification. For peak the list generation, database searching, and FDR estimation, Agilent Spectrum Mill MS proteomics Workbench B.05 was employed. The male MS proteomics data, which we have generated previously, used for this study were taken from ProteomeXchange Consortium (http://proteomecentral.proteomexchange.org) via the PRIDE partner repository with the dataset identifier (PXD006907).

### 2.5. Systems Biology Analysis

Cellular Compartments (CC), Biological Processes (BP), and pathways analysis were conducted for the systems biology analysis. The lists of all SNO-proteins was uploaded into MetaCore from Thomson Reuters (MetaCore™ version 6.34 build 69200) and into the Database for Annotation, Visualization and Integrated Discovery (DAVID) Bioinformatics Recourses (version 6.8, https://david.ncifcrf.gov) [35]. Functional annotation tool in DAVID was used for GO terms and KEGG pathway enrichment analysis [35]. The Benjamini–Hochberg correction [36] was used to calculate the *p* value and generate FDR, and terms with FDR values below 0.05 were accepted. The search tool for the interacting proteins (STRING, version 10.0) was used to analyze BP and pathway enrichment (http://string-db.org) [37]. MetaCore from Thomson Reuters (MetaCore™ version 6.34 build 69200) was used for the networks generation after submitting the lists of SNO-proteins. For this, we also used Benjamini–Hochberg correction [36] to calculate the *p* value and generate FDR. The processes/terms with the FDR values of below 0.05 were included.

### 2.6. Ethic Items

All methods were carried out in accordance with the Hebrew University guidelines and regulations. The animals’ experiments were done in accordance with the Institutional Animal Care and Use Committee (Israel) and the Association for Assessment and Accreditation of Laboratory Animal Care International. All experimental protocols for using animals were approved by the Ethics committee of the Hebrew University (approval code: MD-19-16049-3, approved on 19 December 2019).

## 3. Results

In order to investigate the significant sex differences in protein S-nitrosylation in the cortices of 6–8-weeks-old mice (the age of puberty), the S-nitroso-proteome in the cortices were mapped using SNOTRAP-based mass-spectrometry. Two groups were studied, female-cortex and male-cortex. This was followed by large scale systems analysis together with bioinformatics in order to dissect the biological processes and pathways that are possibly affected by S-nitrosylation. Further, we conducted a large-scale quantitative analysis of the shared SNO proteins between female and male cortices to better understand the quantitative changes associated with sex differences.

### 3.1. Sex Differences in S-Nitrosylation in the Mouse Cortex

**Figure 1:**
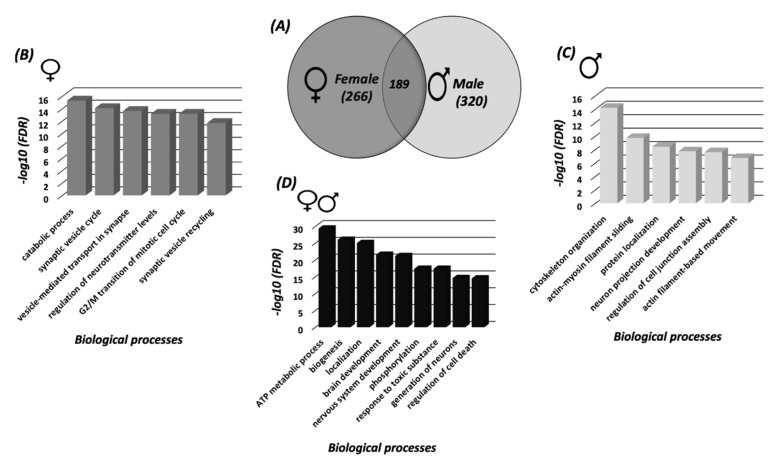
Systems biology analysis of the SNO proteins (**A**) Venn Diagram representing the SNO-proteins that were identified in female cortices and male cortices. BP analysis was conducted on the SNO proteins that are exclusive to (**B**) female cortices, (**C**) male cortices and (**D**) the shared SNO proteins. Each bar represents the −log10 of the False discovery rate (FDR) value.

Mapping the S-nitroso-proteome in female and male cortices using SNOTRAP technology revealed differences between the female and male cortices in mice. Different sets of proteins that underwent S-nitrosylation were identified in both female and male samples. A total of 266 SNO proteins were exclusive to females, a total of 320 SNO proteins were exclusive to male, and a total of 189 were shared between female and male samples (Figure 1A). The detailed lists of the SNO proteins of each group are presented in Supplementary Table S1.

### 3.2. Systems Biology Analysis of the SNO Proteins in the Cortices of Female and Male Mice

In order to investigate the sex differences in biological processes and obtain systems-level insight into SNO-functionalities, we performed a large-scale systems analysis by analyzing the biological processes (BP), molecular functions (MF), and cellular components (CC).

In the female cortex, the BP analysis revealed significant enrichment of synaptic and mitotic processes; such as synaptic vesicle cycle (FDR = 8.32 × 10^−15^), vesicle-mediated transport in synapse (FDR = 2.4 × 10^−14^), G2/M transition of mitotic cell cycle (FDR = 6.79 × 10^−14^) and others (Figure 1B). The MF analysis showed a significant enrichment of nitric-oxide synthase binding (FDR = 0.000183), ATPase activity (FDR = 9.04 × 10^−5^), channel regulator activity (FDR = 0.000715) and others (Supplementary Figure S1A). CC analysis showed enrichment of myelin sheath (FDR = 1.36 × 10^−27^), cytosol (FDR = 1.37 × 10^−22^), synapse (FDR = 1.06 × 10^−13^) and others (Supplementary Figure S2A).

In the male cortex, the BP analysis demonstrated significant enrichment of cytoskeletal and actin-related processes such as cytoskeleton organization (FDR= 6.09 × 10^−15^), actin-myosin filament sliding (FDR = 1.93 × 10^−10^), protein localization (FDR = 3.86 × 10^−9^) and others (Figure 1C). The MF analysis showed enrichment of cytoskeletal protein binding (FDR = 3.00 × 10^−15^), structural constituent of cytoskeleton (FDR = 0.000244), actin binding (FDR = 0.000294) and others (Supplementary Figure S1B). CC analysis showed enrichment of cytoskeleton (FDR = 6.01 × 10^−16^), cytosol (FDR = 5.39 × 10^−13^), axon (FDR = 4.03 × 10^−12^) and others (Supplementary Figure S2B). The detailed lists of the BP, MF and CC enriched in both female and male cortex can be found in Supplementary Tables S2 and S3, respectively.

Furthermore, in order to investigate the shared processes and mechanisms between both sexes, BP analysis was conducted to identify the SNOed proteins common to both female and male cortices. The analysis indicated a significant enrichment of processes that are functionally related to mitochondria and neurodevelopment, such as ATP metabolic processes (FDR = 4.6 × 10^−30^), nervous system development (FDR = 7.31 × 10^−22^), generation of neurons (FDR = 2.45 × 10^−15^) and others (See Figure 1D).

**Figure 2:**
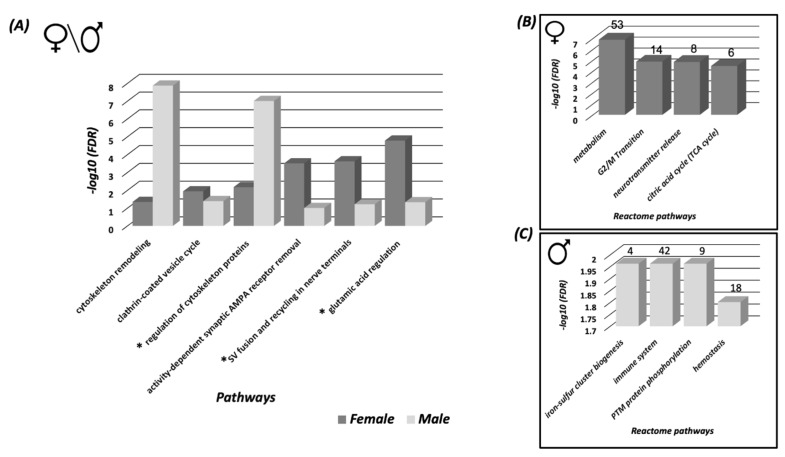
Pathways and interactome analysis of the exclusive SNO proteins in the female and male cortices. (**A**) Pathways analysis of the SNO proteins that are exclusive to female and male cortices. Reactome pathway analysis was conducted on the SNO proteins that are exclusive to (**B**) the female cortex and (**C**) the male cortex. The number of the SNO proteins in each pathway is presented above each bar. Each bar represents the −log10 of the FDR value. Abbreviations: * Regulation of cytoskeleton proteins in oligodendrocyte differentiation and myelination, * Synaptic vesicle fusion and recycling in nerve terminals, * Glutamic acid regulation of Dopamine D1A receptor signaling.

**Figure 3:**
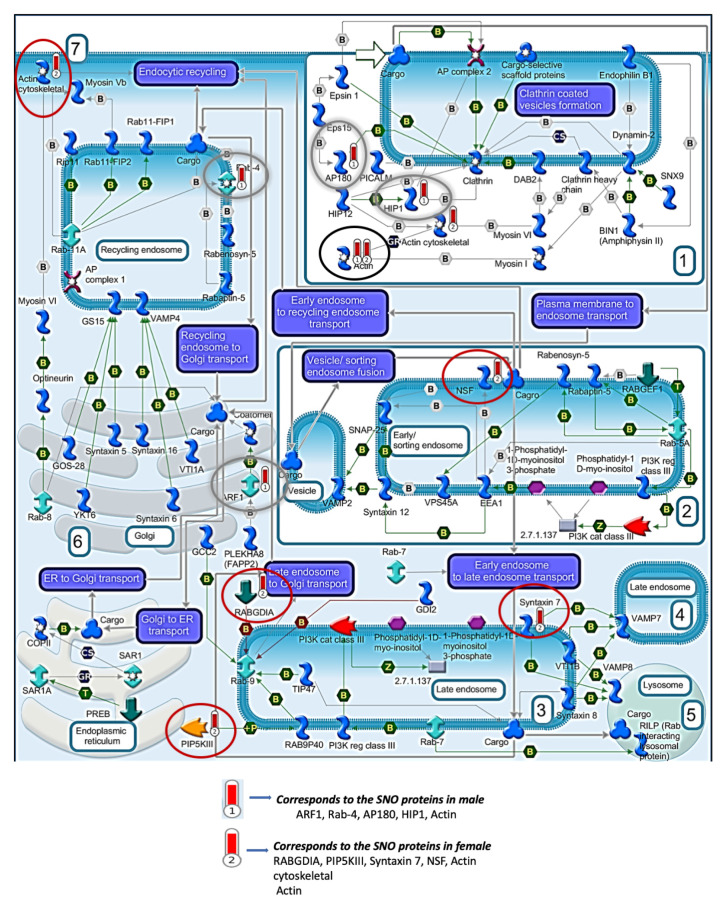
“Clathrin-coated vesicle cycle” pathway was enriched in both sexes. Red circles are SNO proteins in female. Gray circles are SNO proteins in male. Black circles are SNO proteins in both female and male groups.

**Figure 4:**
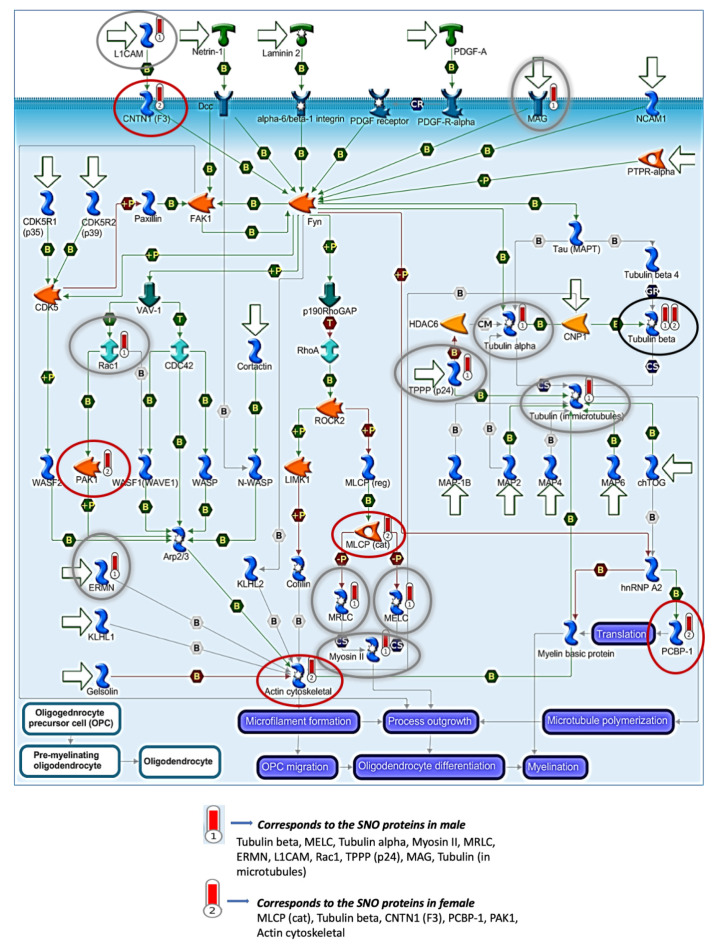
“Regulation of cytoskeleton proteins in oligodendrocyte differentiation and myelination” pathway was enriched in both sexes. Red circles are SNO proteins in female. Gray circles are SNO proteins in male. Black circles are SNO proteins in both female and male.

Pathways analysis of the SNO proteins of each tested group was conducted in order to test the shared signaling pathways that are affected and controlled by SNO in both sexes. The pathways analysis was consistent with the BP analysis of the females and males and showed significant involvement of the SNO proteins in synaptic and cytoskeleton-associated pathways (Figure 2A). For example, “Clathrin-coated vesicle cycle” pathway was significantly enriched with FDR = 0.01146. The “regulation of cytoskeleton proteins in oligodendrocyte differentiation and myelination“ pathway was significantly enriched with FDR = 9.8 × 10^−8^. It is worth mentioning that despite both pathways were enriched in both sexes, different proteins were involved in the signaling regulation. The detailed cell trafficking of both signaling pathways are shown in Figure 3 and Figure 4.

To test the pathways affected by proteins networks, reactome pathways analysis was performed. Reactome pathway refers to the networks of biological and molecular interactions by which the proteins clusters can form. In the female cortex, the enriched reactome pathways were consistent with the BP and pathways analysis. For example, “Metabolism” (*n* = 53, FDR = 1.36 × 10^−7^), “Neurotransmitter release cycle” (*n* = 8, FDR = 1.43 × 10^−5^), and others (Figure 2B). In the male cortex, the analysis revealed significant enrichment of “immune system” (*n* = 42, FDR = 0.0109), “Post-translational modification (PTM) protein phosphorylation” (*n* = 9, FDR = 0.0109), and others (Figure 2C).

### 3.3. Protein Classification Analysis of the SNO Proteins in Female and Male Cortices

In order to examine the functions of SNO-proteins, the protein classification analysis was carried out. We identified diverse protein families which can be targeted by the S-nitrosylation, such as proteases, enzymes, ligases, kinases, and others (Supplementary Figure S3) and determined the IDs of the SNO-related kinases and phosphatases in the cortices of mice (Supplementary Figure S4).

### 3.4. Quantitative Analysis of the S-Nitroso-Proteome in Both Sexes

To promote deep understanding and to visualize the quantitative differences in SNO proteins in the female and male cortices, we performed a large-scale quantitative analysis. A total of 189 SNO proteins were detected in both females and males were detected.

**Figure 5:**
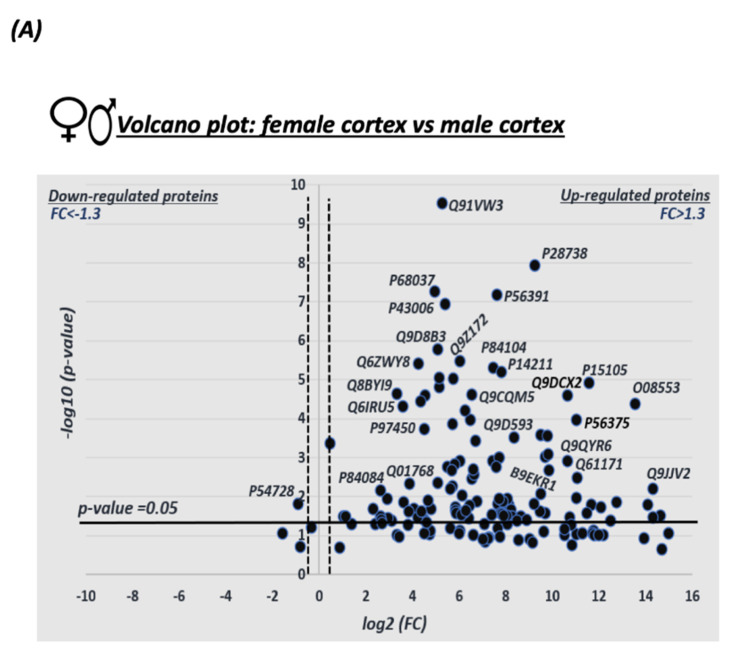

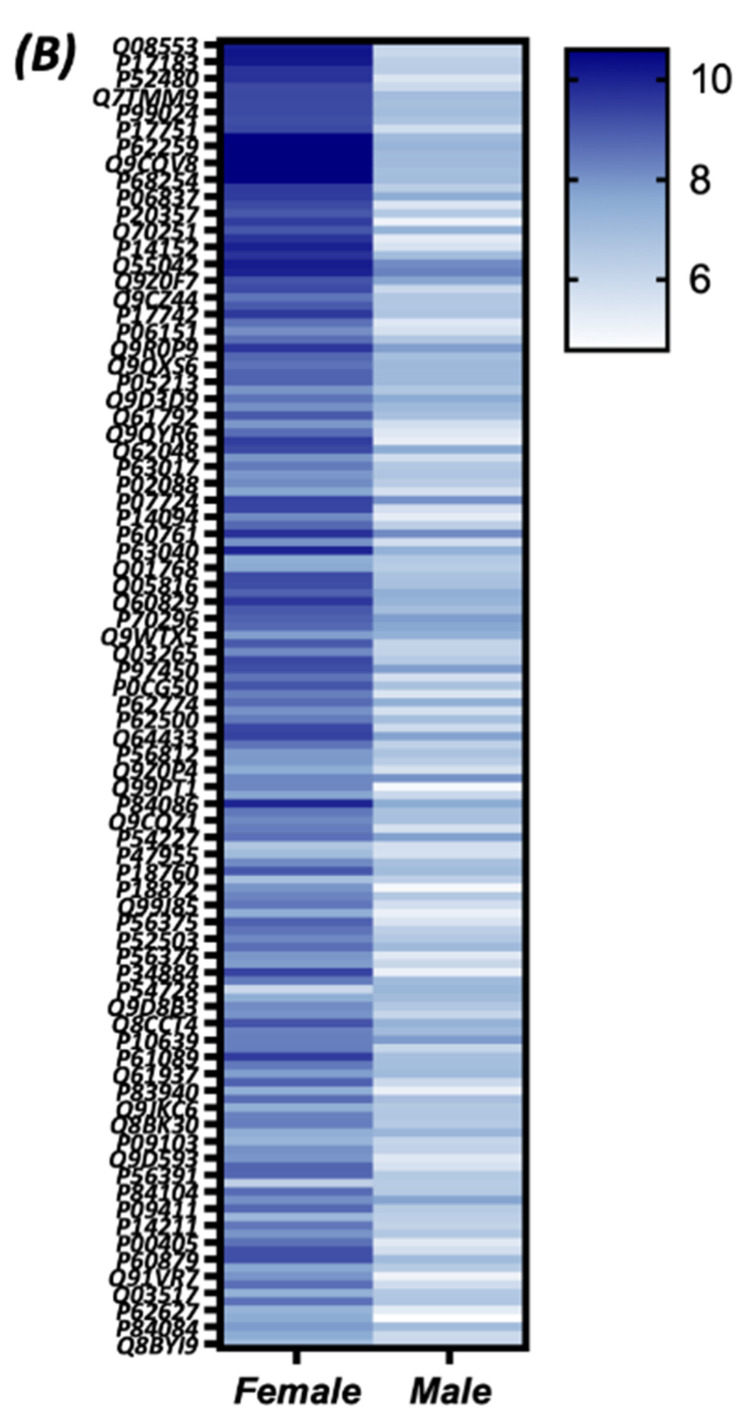
Quantitative analysis of the shared SNO-proteins between female and male cortices. (**A**) Volcano Plot analysis was conducted on the shared SNO proteins in the male cortex *vs*. the female cortex. The X axis represents the log2 of the fold change (FC)), that was calculated as the difference in the relative abundance of each protein in both female and male divided by the relative abundance of the protein in males. FC $=\frac{\mathrm{Relative abundnce}(F)-\mathrm{Relative abundance}(M)}{\mathrm{relative abundance}(M)}$. The Y axis represents the *p*-value. The horizontal line represents a significance level of *p* = 0.05. The vertical lines represent the threshold of the FC = 1.3. Proteins with “relative high abundance” in female are those that appear on the right side of the plot with statistical significance of *p*-value < 0.05 and FC > 1.3. (**B**) Heat map analysis representing the differential relative abundance of the shared SNO-proteins in the female and male cortices. Each line represents one protein identified by its accession ID. The relative abundance scale was normalized by –log10.

Volcano plot analysis was conducted in order to visualize and identify the significant changes in the expression of the SNO proteins in both female and male mice (Figure 5A). The X axis represents the fold change (log_2_(FC)) that was calculated as the difference in the protein’s relative abundance in both female and male cortices divided by the protein’s relative abundance in the male cortex (as an initial state) and plotted against the Y axis that represents *p*-values (−log_10_*p*-value). Statistically significant thresholds of FC > 1.3 and *p* < 0.05 indicate significantly upregulated SNO proteins in females. The thresholds of FC < −1.3, *p* < 0.05 correspond to significantly downregulated proteins in females. Strikingly, the analysis revealed a total of 111 proteins that were significantly more abundant in the female cortex (see Figure 5A).

To further visualize the quantitative differences in the relative abundance of the SNO proteins in the female and male cortices, we conducted a heat map analysis that shows the high intensity of the SNO-proteins in the female *vs*. the male group (Figure 5B).

## 4. Discussion

We used the state-of-the-art proteomics and bioinformatics techniques to study the sex differences in biological processes related to protein S-nitrosylation in the cortices of juvenile wild type (6–8-weeks-old) mice. S-nitrosylation occurred to a large variety of proteins belonging to different pathways and responsible for different biological processes in the brain. However, the results of our work showed, for the first time, significant differences in the extent and the targets of protein S-nitrosylation in male and female mice, which bring about the sex differences in the signaling pathways and biological processes.

266 proteins were S-nitrosylated (SNOed) exclusively in the female cortex, 320 proteins were SNOed exclusively in the male cortex, and 189 proteins were SNOed in both male and female mice (Figure 1A). The GO analysis in the cortices of female mice revealed the SNO-related significant enrichment of the synaptic and mitotic processes, such as synaptic vesicle cycle, vesicle-mediated transport in synapses, G2/M transition of mitotic cell cycle, etc. (Figure 1B).

All brain functions are mediated and controlled by the synapses, which provide the contacts between neurons and, often, dendrites and axons [38]. On the other hand, it is now appreciated that synapse formation is promoted by the brain activity [39]. Importantly, it has been found that NO is involved in regulation of the synaptic processes by the brain. Indeed, NO can induce SNO of total protein in synaptosomes [40], implying that this molecule may contribute to the regulation of synaptic protein functions. Thus, it has been shown that the NO donor nitrosoglutathione (GSNO) can increase SNO of vesicular monoamine, vesicular acetylcholine and vesicular glutamate transporters in the mouse brain. Furthermore, GSNO also induces an inhibitory effect on the vesicular uptake of these mediators [40]. The ability of NO to modulate the release of other neurotransmitters, such as GABA and dopamine (DA) has also been confirmed [41,42]. In experiments on rats, it has been demonstrated that the NO donor S-Nitroso-*N*-acetyl-dl-penicillamine increased glutamate release in the hippocampus [43] and medulla oblongata [44], whilst NOS blockers totally blocked *N*-methyl-d-aspartate (NMDA)-induced glutamate release in the hippocampus [45]. NO can also stimulate cGMP-dependent GABA release in different brain areas [40,46]. The role of NO in NMDA-dependent DA release has also been confirmed in other CNS regions such as the lateral olivocochlear nuclei [47] and the medial preoptic area [48]. On the other hand, the inhibitory effect of NO on GABA release has been shown in the internal granule cell layer of the cerebellum and auditory cortical neurons [40]. Serotonin release is controlled by NO in different CNS regions, and it has been hypothesized that this molecule represents an important transmitter coupling glutamatergic to serotoninergic neurotransmission [40]. Interestingly, in experiments on the granule cells of the cerebellum, Ho et al. have demonstrated that NMDA-mediated production of NO targets PSD-95 (a principal scaffolding component of the postsynaptic density) to synapses via SNO, which competes with another kind of PTM of cysteine, palmitoylation [49]. Taken together, these data indicate the significance of the SNO-related enrichment of the synaptic processes because NO and SNO play a central role in the regulation of vesicular neurotransmitter transport and development of the synaptic structures [50].

Importantly, the results of this work show that the NO and SNO-related synaptic processes differ between the two sexes in mice. In fact, these processes were significantly enriched in the female cortex and this was not observed in their male counterparts. The cellular mechanisms regulating sexually dimorphic synaptic patterning remain elusive [51]. It has been found that circulating estrogens increase astrocytic surface area, such that astrocyte processes ensheath of the neurons. This results in an increase in synapse formation in female arcuate nucleus in the hippocampus compared to the male one [51]. It is well known that estradiol can potentiate excitatory synapses in the hippocampus [52,53,54], and attenuate a perisomatic inhibitory effect on synapses in females but not in males [55]. Furthermore, it has been shown that estrogen can influence memory formation by enhancing synaptic strengthening [56] and regulating synapse numbers [57] in the hippocampus. Remarkably, these estrogen effects were described in the female, but not in male mice [58]. It has also been found that estradiol can strengthen long-term potentiation (LTP) through activation of estrogen receptor β signaling, possibly by inducing signaling through the tyrosine kinase in the hippocampus [59], prefrontal [60,61] and sensory-motor cortex [62]. The above data support our finding on the preferential enrichment of the synaptic processes in females vs. males. However, to the best of our knowledge, the role of NO and SNO in the sexual dimorphism of synaptic processes in the brain has not been reported previously. Considering the role of synapses in the brain functions, it is obvious that the enrichment of the synaptic processes in females may have a significant impact on a variety of brain functions in physiological and pathological conditions.

The MF analysis of this study revealed that SNO in female mice significantly enriched NOS binding (Supplementary Figure S1A). These results are in accord with previously published data indicating the ability of estrogen to stimulate nNOS [19,20,21,22]. For example, Edelman et al. have found that females have significantly more nNOS in the developing hypothalamus and preoptic area when compared with males [19].

In contrast to the female cortex, the SNO proteins in the male cortex demonstrated a pronounced enrichment of cytoskeletal processes, such as cytoskeleton organization, actin-myosin filament sliding, actin filament-based movement and others (Figure 1C). Similarly, the MF and CC analysis showed enrichment of cytoskeleton, e.g., cytoskeletal protein binding, structural constituent of cytoskeleton, actin binding, etc. (Supplementary Figure S1B). Cellular functions are tightly associated with the cytoskeleton, and NO is an important regulator of these functions, including cell motility, shape, contraction, and mitosis. The accumulating data show that the SNO of cytoskeletal proteins selectively alters their function. It complements the effects of cGMP on cytoskeletal proteins in the cells, modulating cell migration and contractility [11]. Accordingly, it has been shown that the proteostasis of actin and tubulin proteins is precisely controlled during neuronal maturation in the brain, and NO signaling appears to be a key regulator of these processes [63]. For example, microtubule-associated protein 1B, an essential regulator of the axonal cytoskeleton [64], has been reported to regulate nNOS-dependent axon retraction, and this process is mediated by SNO of the light chain of this protein at Cys-2457 [65]. Functions of actin polymerization [66,67], members of the cofilin family [68,69], α and β spectrin [70], α- and β-tubulin [71,72], kinesin [73,74], myosin [75] and other cytoskeletal proteins are all affected by SNO. Therefore, the SNO-induced enrichment of cytoskeletal processes found in our study is not surprising. However, our results point to the increased enrichment of these processes in male as compared to female mice.

The data on the sex differences in regulation of the cytoskeleton are equivocal. On the one hand, Hansberg-Pastor et al. have reported that estradiol and progesterone promote the remodeling of the actin and dendrite cytoskeleton [76], induce morphological changes in shape, size, and number of neuronal spines [77]. On the other hand, it is evident that testosterone is also involved in the development and support of the cytoskeletal proteins in the brain. For instance, it has been found that castration of adult male rats reduces the dendritic length and soma size of the neurons, and these changes can be reversed by androgen treatment [78,79]. In the experiments on human neuroblastoma SH-SY5Y cells, it has been found that testosterone resulted in an up-regulation of α- and β-tubulin and this effect was attenuated by co-incubation of testosterone with antiandrogens. The authors concluded that tubulin is a direct neuronal target of androgen regulation [80]. Our results indicate that in male mice, the cytoskeletal processes are more dependent on the SNO signaling than in females. This is a novel finding and the physiological significance and the mechanism of this phenomenon have yet to be discovered.

S-nitrosylation can target a variety of proteins such as kinases, phosphatases, proteases and others, and this was confirmed in our study (Supplementary Figures S3 and S4). This kind of PTM affects the activity of these enzymes, inducing predominantly inhibitory effect both in normal and pathological conditions [12,81,82]. Changes in the activity of the enzymes due to SNO result in modulation of the activity of their downstream targets and activation or inhibition of the adjacent signaling pathways.

In this study, SNO enriched the metabolic, in particular, mitochondrial processes, such as ATP-related processes (Figure 1D), in both male and female mice. This finding corresponds to the data of other researchers. Thus, it has been found that NO can be produced in the mitochondria by nNOS [83] or diffuse into these organelles at a short distance from its site of generation by iNOS [84,85]. It has been previously confirmed that a wide variety of mitochondrial proteins can be subjected to SNO, including the proteins involved in the citric acid cycle, electron transport chain, fatty acid oxidation [86,87], mitochondria fission [88] etc., modulating the activity of these processes [89].

The analysis of the MS data revealed 189 proteins which were significantly SNOed both in males and females. To visualize the quantitative differences in the expression of these shared proteins, the volcano plot with a heat map was generated (Figure 5). Strikingly, the volcano plot showed that among these proteins, a total of 111 proteins were more abundant in the female cortex. On the other hand, a higher number of proteins were exclusively SNOed in males compared to females. However, further studies are needed to examine the levels and activity of nNOS and to get a cut conclusion whether NO levels are different between sexes.

NO occupies a special role in regulation of numerous brain functions acting as an important signaling molecule. However, overproduction of NO promotes the formation of peroxynitrite [14], protein SNO, tyrosine nitration and formation of GSNO [15,16,17,18] which are involved in pathogenesis of a variety of neurodevelopmental and neurodegenerative brain disorders [90]. In the experiments on mice carrying *Shank3* mutation (a model of autism spectrum disorder (ASD)), we have recently found dramatic changes in S-nitrosylation of proteins responsible for the synaptic vesicle cycle, neurotransmission and neurodevelopment in the cortices of these mice [91]. Dysregulation of all these processes and pathways are involved in the pathogenesis of ASD [92]. It is well established that ASD affects more male than female children with the ratio of 4:1 [93]. This difference could be partially related to the fact that the synaptic processes are more enriched in females than in males, according to the results of this study and the data of others [51,52,53,54]. Alzheimer’s disease (AD) is the most common chronic neurodegenerative disorder [94]. A large body of evidence indicate that NO and SNO are involved in the pathogenesis of this disease. Experiments on the CK-p25 mouse model of AD [33], cultured cortical neurons [95], and post-mortem studies [96,97,98,99] have convincingly shown the accumulation of SNO proteins which are directly involved in the development of AD. This brain disorder prevails in elderly women as compared to old men [100]. Vina and Lloret have explained this phenomenon by the protective effect of estrogen against amyloid-β toxicity, oxidative stress, and release of apoptogenic signals in mitochondria [101]. Importantly, NO is involved in this protective effect of estrogen [102]. However, the level of this hormone is reduced in old females placing them at a higher risk of developing AD [101]. Therefore, the differences in NO production and SNO protein profiles between males and females may affect the risk and the course of developing the neurodevelopmental or neurodegenerative diseases and these differences need to be taken into account while elaborating new effective pharmacological strategies for treating these patients.

In conclusion, a number of proteins were exclusively SNOed in the cortices of female mice, others in those of male mice, and some proteins were subjected to this PTM in mice of both sexes. Analyzing these proteins, we found—for the first time—significant sex differences in the protein S-nitrosylation in the cortices of mice and in the biological functions of the brain related to SNO. These differences are as follows:

(1) Significant SNO-induced enrichment of the synaptic processes was found in female mice; (2) significant SNO-induced enrichment of NOS binding was also observed in female cortex; (3) the SNO-dependent enrichment of cytoskeletal processes was considerably higher in the male cortex; (4) shared signaling pathways found in both sexes appeared to be modulated by different SNO-proteins.

Thus, our MS analysis based on the large-scale computational biology revealed significant sex differences in the protein S-nitrosylation and in the biological functions controlled by this PTM. To the best of our knowledge, these sex differences have not been previously attributed to SNO. The results of this study point to the sex dimorphism in the SNO-related regulation of the brain functions, particularly in the synaptic, cytoskeletal, metabolic and NOS processes. They provide a mechanistic understanding of the role of NO and S-nitrosylation in females and males. At the same time, further investigations are needed to unravel the intrinsic mechanisms of the NO-related regulation of brain functions in males and females.

## Supplementary Materials

The following are available online at https://www.mdpi.com/2227-9059/8/5/124/s1, Figure S1. MF analysis was conducted on the SNO proteins that are found exclusively in the (A) female cortex and (B) male cortex groups. Each bar represents the −log10 of the FDR value. Figure S2. CC analysis was conducted on the SNO proteins that are found exclusively in the (A) female cortex and (B) male cortex groups. Each bar represents the −log10 of the FDR value. Figure S3. Proteins’ classification analysis of the SNO proteins that are found exclusively in the (A) female cortex and (B) male cortex groups. Figure S4. the SNOed kinases and phosphatases in the (A) female cortex and (B) male cortex groups. Table S1. IDs of SNO-proteins in the different groups. Table S2. System biology analysis of the female cortex group. Table S3. System biology analysis of the male cortex group.
