# Supplementary material for: Sex Differences in Biological Processes and Nitrergic Signaling in Mouse Brain
Source: Biomedicines. 2020 May 15;8(5):124. doi: 10.3390/biomedicines8050124 (PMC7277573; doi:10.3390/biomedicines8050124)
Supplement: Supplementary file 1 [file biomedicines-08-00124-s001.zip › biomedicines-775600-supplementary.docx]

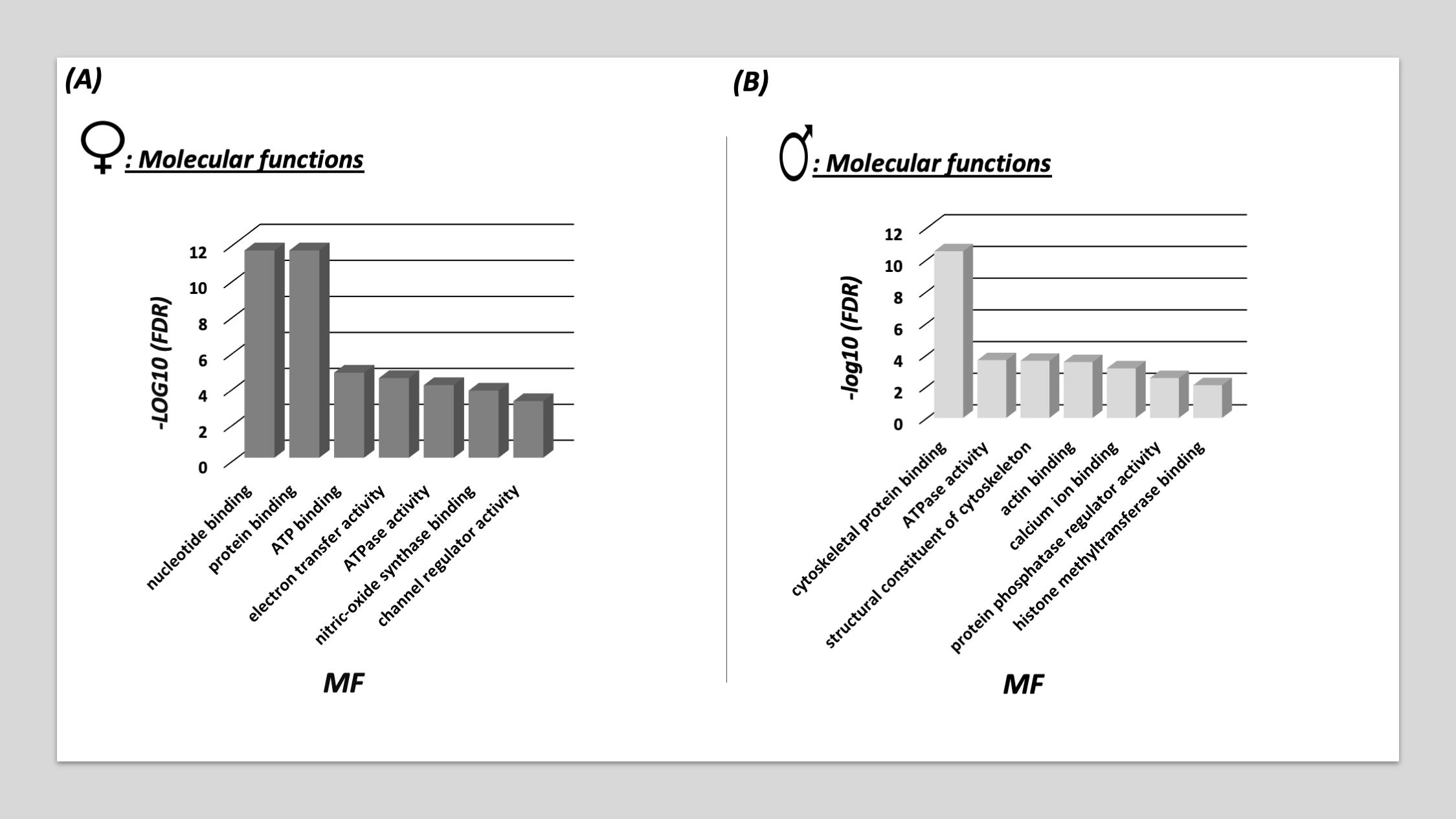


**Figure S1.** MF analysis was conducted on the SNO proteins that are found exclusively in the (A) female cortex and (B) male cortex groups. Each bar represents the −log10 of the FDR value.


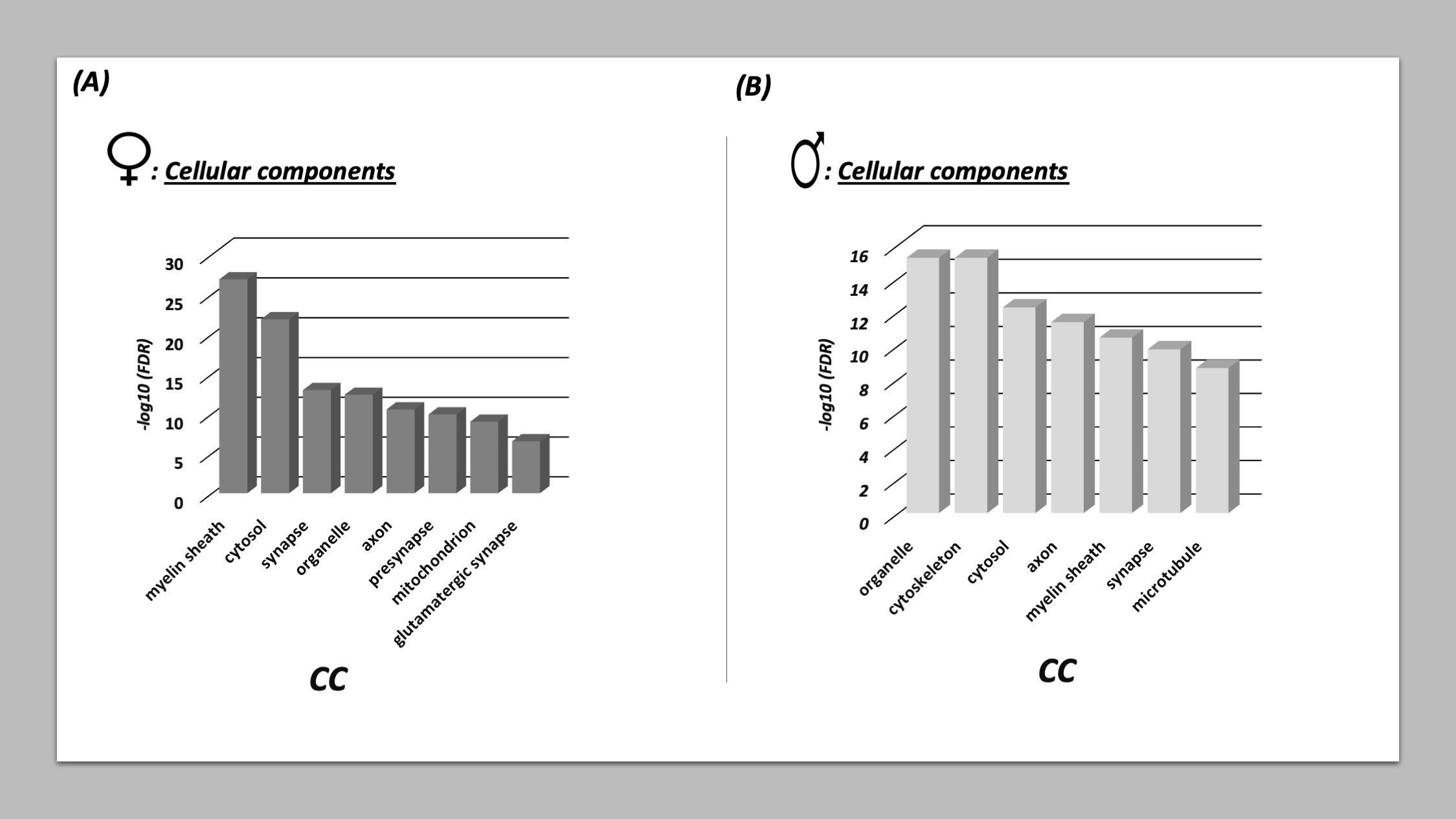


**Figure S2.** CC analysis was conducted on the SNO proteins that are found exclusively in the (A) female cortex and (B) male cortex groups. Each bar represents the −log10 of the FDR value.


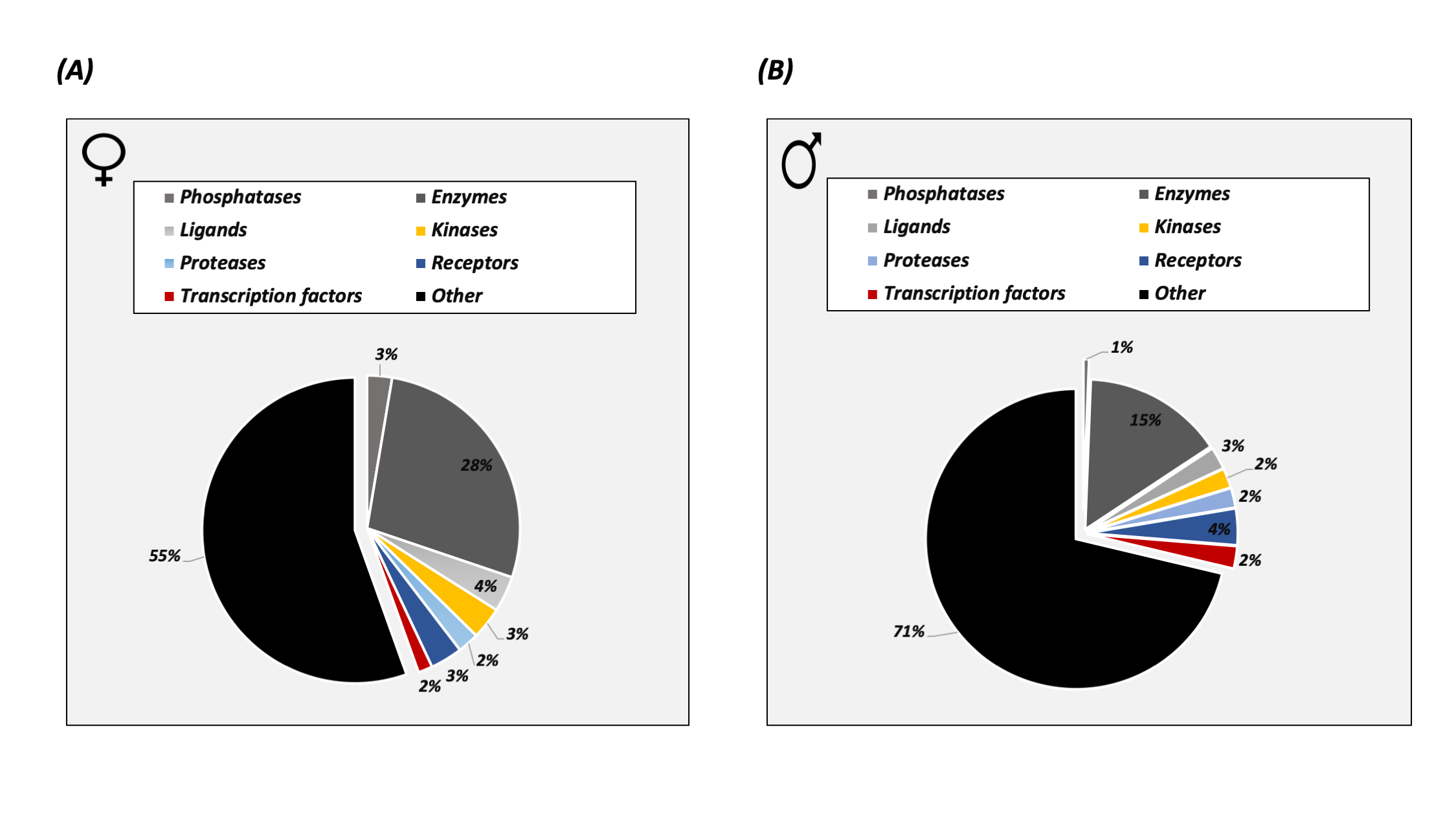


**Figure S3.** Proteins’ classification analysis of the SNO proteins that are found exclusively in the (A) female cortex and (B) male cortex groups.

***
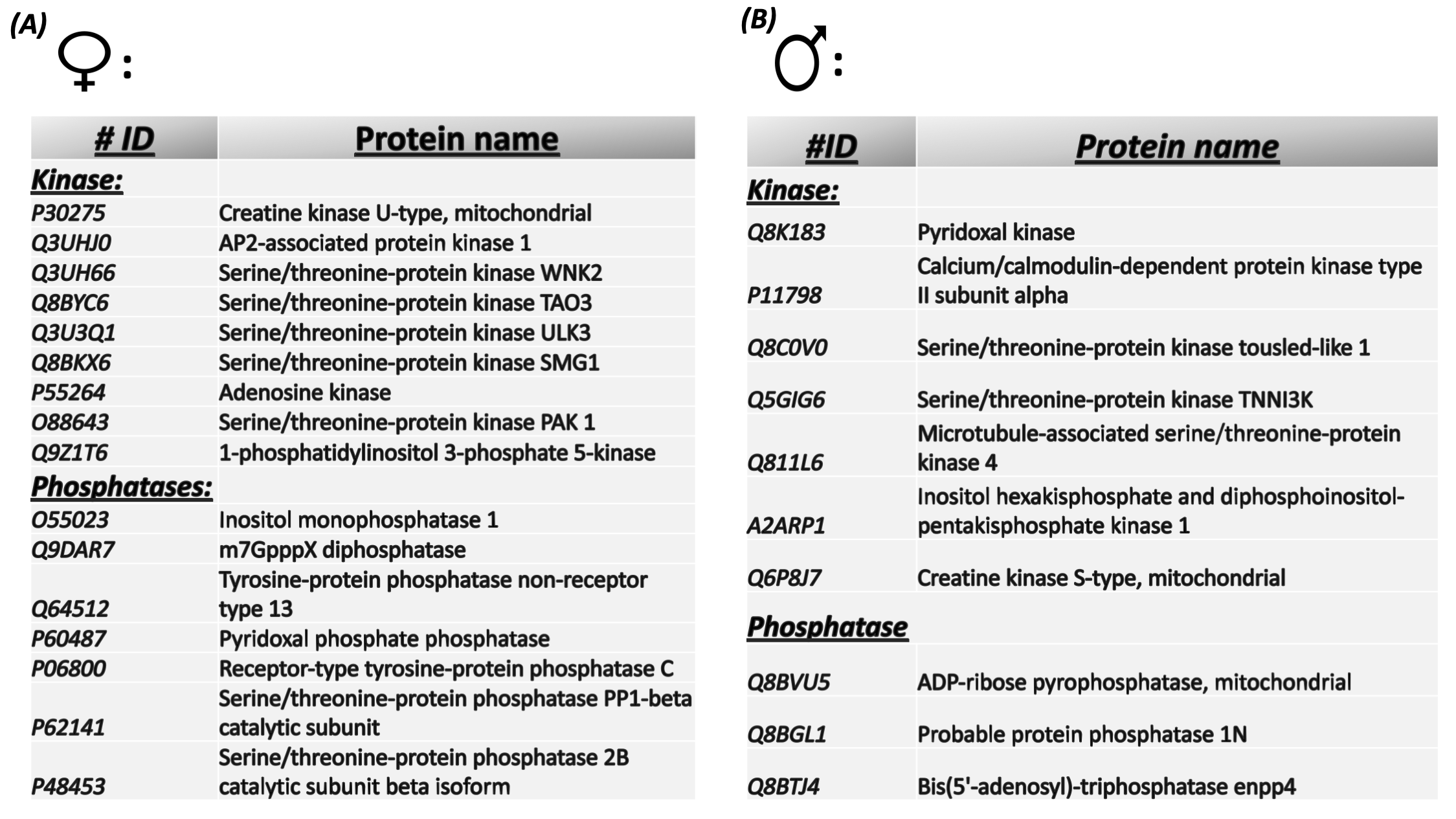
***

**Figure S4.** the SNOed kinases and phosphatases in the (A) female cortex and (B) male cortex groups.

**Supplementary Tables are uploaded as Excel files:**

**Table S1.** IDs of SNO-proteins in the different groups.

**Table S2.** System biology analysis of the female cortex group.

**Table S3.** System biology analysis of the male cortex group.
